# Regression of right ventricular systolic pressure after successful percutaneous mitral commissurotomy in patients with isolated severe mitral stenosis

**DOI:** 10.12669/pjms.333.12565

**Published:** 2017

**Authors:** Shah Zeb, Tariq Ashraf, Muhammad Hashim, Syed Nadeem Hassan Rizvi

**Affiliations:** 1 Shah Zeb, MRCP, FCPS. Department of Interventional Cardiology, National Institute of Cardiovascular Disease, Karachi, Pakistan; 2 Tariq Ashraf, FCPS, FACC. Department of Interventional Cardiology, National Institute of Cardiovascular Disease, Karachi, Pakistan; 3 Muhammad Hashim, FCPS, FACC. Department of Interventional Cardiology, National Institute of Cardiovascular Disease, Karachi, Pakistan; 4 Syed Nadeem Hassan Rizvi, FRCP, FCPS, FACC, SCAI Fellowship. Department of Interventional Cardiology, National Institute of Cardiovascular Disease, Karachi, Pakistan

**Keywords:** Percutaneous transluminal mitral commissurotomy (PTMC), Right ventricular systolic pressure (RVSP)

## Abstract

**Objective:**

To know the regression of right ventricular pressure after successful percutaneous transluminalmitral commissurotomy (PTMC) in patients with severe isolated mitral stenosis.

**Methods:**

This descriptive study was performed in inpatient and outpatient department of National Institute of Cardiovascular Disease from 1^st^ February 2016 to 31^st^ August 2016. Echocardiography of all patients with successful PTMC were recorded 24 hours and 06 months following PTMC to see for Regression of right ventricular pressure along with other baseline echocardiographic parameters.

**Results:**

A total of 99 patients with severe isolated mitral stenosis who had undergone successful PTMC were studied. Females were 65(65.7%) and males 34(34.3%). Mean age was 27.44±6.26 years. TTE performed before and after PTMC showed significant difference in mean mitral valve area (0.89cm ±0.089cm2 vs. 1.68±0.128 cm2, p valve <0.001) and mean left atrial diameter (4.66± .82cm vs. 4.46± 0.65cm). Mean mitral valve gradient pre PTMC was significantly higher (16.38±2.51 mm of Hg) than that of post PTMC 24 hours (4.75±1.31 mm of Hg) and Post PTMC 06 months (5.22±1.21 mm of Hg), p valve <0.001. Mean right ventricular systolic pressure (RVSP) pre PTMC was significantly higher 62.3±10.91 mm of Hg than that of post PTMC 24 hour’s 57.51±9.67 mm of Hg and post PTMC 06 moths 46.49±7.8mm of Hg, p value 0.001. Mean LVEF 50.14± 5.82.

**Conclusion:**

There was a significant regression of right ventricular pressure following successful PTMC in mid-term (06 months) follow up of severe isolated mitral stenosis patients.

## INTRODUCTION

Rheumatic mitral stenosis is one of the commonest valvular heart lesions in developing countries.^1^ Severe mitral stenosis causes increase in the backflow pressure in the pulmonary vasculature which causes pulmonary venous hypertension. Percutaneous transluminal mitral commissurotomy (PTMC) has become the treatment of choice for patients with symptomatic rheumatic mitral stenosis (MS). Several studies have reported good immediate, short-term and long-term results.^2^-^4^

Right ventricular function is important in the development of patient symptoms and prognosis in mitral stenosis patients. Right ventricular dysfunction in this category of patient may result from direct affection of the myocardium by the rheumatic process or secondary to hemodynamic alterations due to pulmonary vascular changes which lead to RV overload and failure.^5^,^6^ The impaired RV function may improve after successful PTMC.^7^,^8^ It is hypothesized that after successful PTMC, The pulmonary artery pressure can be regress after mid to long term follow up following successful PTMC.

Since rheumatic heart affection is more severe and the degree of valvular damage is greater in developing countries than in industrialized Western communities, it seems appropriate to examine it in this part of the world. Our objective was to determine eregression of right ventricular pressure after successful percutaneous mitral commissurotomy (PTMC) in patients with severe isolated mitral stenosis.

## METHODS

This descriptive study was conducted from 1^st^ February 2016 to 31^st^ July 2016 in Interventional Cardiology Department of National Institute of Cardiovascular Diseases, Karachi. Patients with severe isolated mitral stenosis after successful PTMC were followed for at least six months. Patients with severe isolated mitral stenosis having undergone successful percutaneous transluminal mitral commissurotomy (PTMC) were included by non-probability consecutive technique. All the patients were evaluated with transthoracic echocardiography (TTE) by a standard technique using Toshiba Xario 2100 echocardiographic machines before PTMC, 24 hours and 6 months after PTMC. Transesophageal echocardiography (TEE) was performed only in patients with atrial fibrillation to rule out left atrial/left atrial appendage thrombus. TTE had been used in this study for measurements of cardiac chamber dimensions and for the assessment of left and right ventricular performance. Severe mitral stenosis was defined by echocardiographic criteria as associated with mean transvalular gradient of more than 10 mm of Hg, pulmonary artery pressures of more than 50 mm of Hg and a valve area of less than 1 cm^2^.

PTMC was done by double (Multitrack) double balloon technique. The procedure was performed by two experienced interventional cardiologist who also monitored the hemodynamics during the case.

Pulmonary artery pressures were measured from Right ventricular systolic pressure plus estimated Right atrial Pressure. Wilkins score was obtained by adding the score for each of the individual morphological features as, leaflets mobility, thickness, calcification and subvalvular lesions. Mitral and tricuspid regurgitation was assessed by color flow mapping. TTE was repeated 24 hours after PTMC to know about the successful PTMC.

Optimal PTMC was defined as post PTMC mitral valve area of ≥1.5cm^2^ or at least 25 % increase in valve area with no more than one grade increase in MR and with no major complication. All the patients with diabetes, hypertension, suboptimal PTMC, mitral regurgitation more than Grade-1, Aortic regurgitation more than grade 1, evidence of coronary artery disease or left ventricle ejection fraction (LVEF) <40% were excluded from the study. The demographic, clinical and echocardiographic variables were entered through a specially designed proforma.

All the data were documented in specially designed proforma for this study. The data was analyzed by SPSS Version 14.0. Continuous variables were expressed as mean ± SD while Categorical variables were expressed as numbers and percentages. Chi square was used for comparing Categorical variable while Student T test was used for comparing numerical variable respectively.

## RESULTS

**Fig.1:**
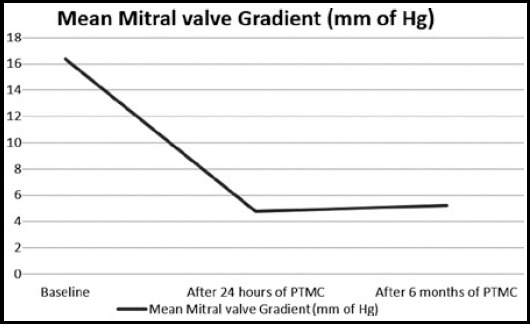
Mean difference in mitral valve gradient before, after 24 hours and after 6 months.

**Fig.2:**
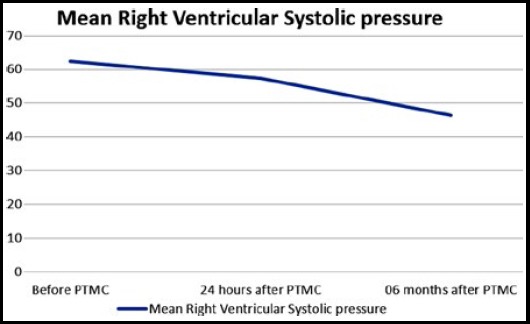
Mean difference in right ventricular systolic pressure before PTMC, 24 hours after PTMC and 6 months after PTMC.

A total of 99 patients, 65(65.7%) female and 34 (34.3%) male with severe isolated mitral stenosis who had undergone successful PTMC were included.. Mean age of patients was 27.44±6.26 years as shown in Table-I. TTE performed before and after PTMC showed significant difference in mean mitral valve area (0.89cm ±0.089cm2 vs. 1.68±0.128 cm2, p valve <0.001), mean left atrial diameter (4.66± .82cm vs. 4.46± .65cm). Mean mitral valve gradient pre PTMC was significantly higher (16.38±2.51 mm of Hg) than that of post PTMC 24 hours (4.75±1.31 mm of Hg) and Post PTMC six months (5.22±1.21 mm of Hg), p valve <0.001 as shown in Fig.1. There was a non-significant change in pre PTMC and post PTMC Atrial fibrillation 25 (25.3%) vs. 21 (21.2%), p value 0.125. Spontaneous Echo Contrast showed significant change in pre PTMC 49 (49.5%) and post PTMC 19 (19.1%), p valve 0.001 as shown in Table-II. Mean right ventricular systolic pressure (RVSP) pre PTMC was significantly higher 62.3±10.91 mm of Hg than that of post PTMC 24 hour’s 57.51±9.67 mm of Hg and post PTMC 06 moths 46.49±7.8 mm of Hg, p value 0.001 as shown in Fig.2. Mean LVEF 50.14%± 5.82. Pre PTMC severe tricuspid regurgitation was present in 85(85.9%), moderate tricuspid regurgitation was present in 14(14.2%)while post PTMC severe tricuspid regurgitation was present in 33(33.3%), moderate tricuspid regurgitation was documented in 60(60.6%) and mild regurgitation was present in six (6.1%), p value <0.001. Pre PTMC mild mitral regurgitation was present in70 (70.7%), moderate regurgitation was present in 5 (5.1%) and rest of patients have no Mitral regurgitation 24 (24.3%). Post PTMC mild mitral regurgitation was present in 64 (64.7%), moderate regurgitation was present in 18(18.2%), Severe MR in 1 (1.0%) and rest of patients have no Mitral regurgitation 16(16.2%), p value <0.001.

**Table-I:** Baseline Characteristics of study population.

*Characteristics*		*No.*	*Percentage (%)*	*P value*
Age		27.44±6.26 years		
Males		34	34.3%	
Females		65	65.7%	
Weight		48.4±7.36 kg		
Height		156.7±12.46cm		
Atrial fibrillation	Pre PTMC	25	25.3%	0.125
	Post PTMC	21	21.2%
LV ejection fraction		50.14%± 5.82		

**Table-II:** Echocardiographic characteristics of Study population.

*Variables*		*Numbers*	*P value*
Mitral valve area	Pre PTMC	0.9cm ±0.089cm^2^	<0.001
	Post PTMC	1.68±0.13cm^2^	
Spontaneous echo contrast	Pre PTMC	49(49.5%)	<0.001
	Post PTMC	19(19.1%)	
LA diameter	Pre PTMC	4.65±0.82cm	<0.001
	Post PTMC	4.46± 0.06 cm	
Mitral valve gradient	Pre PTMC	16.38± 2.51 mm of Hg	<0.001
	24 hours post PTMC	4.75± 1.31mm of Hg	
	06 months Post PTMC	5.22±1.21 mm of Hg	
Right ventricular diastolic diameter	Pre PTMC	22.59± 4.09 mm	<0.001
	Post PTMC	20.1± 1.7 mm	
Pre PTMC RVSP	Pre PTMC	62.34±10.98 mm of Hg	<0.001
	24 hours post PTMC	57.51± 9.67 mm of Hg	
	06 months Post PTMC	46.49±7.83 mm of Hg	

## DISCUSSION

The present study has showed that majority of patients have a decrement in Right ventricular systolic pressure after successful PTMC.^9^,^10^ Hamdy et al found in their study that up to 61.54% of patients normalized their RV systolic function after successful PTMC which support our findings.^9^ In another study, Mohan, et al.^10^ found that about 65% of patients improved their systolic function within one year duration and their RV systolic function returned to normal values.^10^ Mohan et al., studied 25 patients with isolated rheumatic mitral stenosis before, immediately after (mean, 40±12 h) and at a mean follow-up of 11.5 months and found that after successful PTMC, right ventricular global function tends to normalize in about two-thirds of the patients.^10^

In a recent study conducted in Turkey by Kaya Z et al right ventricular systolic function improved in the early period, and this improvement was seen to continue in the late period of PTMC, they also mentioned that diastolic function did not improve in the early period, but improved in the late period while the right atrium systolic function in the late period was similar to the basal levels.^11^

Right ventricular (RV) function plays an important role in the development of clinical symptoms, exercise capacity, prognosis, and survival in patients having MS.^12^,^13^ Impaired right ventricle function secondary to chronic pulmonary hypertension, is accepted as an undesired and important result of mitral stenosis. The effect of successful PTMC on right ventricular systolic function has not been well studied. Echocardiography, Tissue Doppler imaging (TDI) and magnetic resonance imaging (MRI) are the methods that are used to evaluate RV functions. Conventional 2-D echocardiography and TDI are potentially appropriate non-invasive techniques and are also less expensive.^14^ Because of the ventricle’s complex trapezoidal anatomy, the quantitative echocardiographic assessment of RV function is difficult. Conventional M-mode, Doppler echocardiography evaluation, and TDI echocardiography, which are used to evaluate LV&RV functions, are preferred because they are less affected by physiologic changes in flow velocities and indicate subclinical functional effects. RV is sensitive to changes in afterload because of smaller mass and higher wall stress.^15^ It was surprising to the researchers that the pulmonary artery pressure decreased after PTMC and this decrease reached the basal level at the one year follow-up.^16^ We follow our study population for up to six months to see for any regression of RV systolic function inspite of successful PTMC.

In our study all the confounders i.e. restenosis, new onset AF in patients with previous normal sinus rhythm and worsening Mitral regurgitation were excluded so that they may not affect our study in terms of right ventricular systolic pressure. However there are some patients whose right ventricular systolic functions either does not improve or continue to progress in spite of successful PTMC, such a phenomenon has been described by Inci S, et al, which conclude that because of irreversible changes in the pulmonary vascular bed in a group of patients with pulmonary hypertension in whom there is a pseudo improvement for a given time due to the decreased post-PTMC after load.^16^ Mahfouz et al demonstrated in there study the long term effect of pulmonary artery stiffness on right ventricular functions and tricuspid regurgitation.^17^ They found that stiffness of pulmonary artery is lower significantly in patients who have regression of tricuspid regurgitation and had permanent improvement in RV function after PTMC. In a histo-morphological study of rheumatic heart disease by Malhotra et al.^18^ described the active rheumatic vasculitis in the intramyocardial braches of coronary arterioles or inactive lesions as characterized by fibrosis and medial hypertrophy. They concluded that these vasculitic changes may affect myocardial function and to some extent explain progressive decline in right ventricular function in spite of successful PTMC.

In a study by Drighil et al^19^ there was right ventricular fractional shortening and improvement in systolic functions, as assessed by the Tei index, after successful PTMC. Similarly this study favors our findings of regression of right ventricular systolic pressure by demonstrating that the significantly increased RV functions in the study population without significant pulmonary HT, and maintained its improved function in the mid-term follow up also. Mahfouz et al^20^ determined a significant decrement in the pulmonary hypertension and increase in TAPSE as a Right ventricular functional assessment in post PTMC follow up. As evident from these literature observations that during acute phase and short to mid-term improvement in right ventricular function and regression of right ventricular systolic pressure, there is still very limited data on the long term effect of successful PTMC on right ventricular function. Although transthoracic echocardiography is reproducible and non-invasive method to evaluate cardiac functions, it should be considered that Right Ventricular functional assessment parameters are not fully independent.

### Study limitations

1. This is single-center nonrandomized, with small sample size.
2. The parameters used to predict RV dysfunction were not independent parameters. MRI and IVA (by echocardiography) which are the best tools for RV function assessment are not used.

Our observational study provides ground for further research on this subject especially in third world countries where rheumatic heart disease is still endemic. Our study demonstrates that Mitral valve corrective surgery or PTMC should be done before severe TR, dysfunction of right ventricle, or occurrence of heart failure.

## CONCLUSION

There was a significant Regression of Right ventricular pressure following successful PTMC in mid-term (06 months) follow up of severe isolated mitral stenosis patients.
